# Intravenous thrombolysis with rt-PA in stroke: experience of the moroccan stroke unit

**DOI:** 10.11604/pamj.2016.24.207.8815

**Published:** 2016-07-08

**Authors:** Naima Chtaou, Lamyae Rachdi, Aouatef El Midaoui, Zouhair Souirti, Nils Wahlgren, Mohammed Faouzi Belahsen

**Affiliations:** 1 Neurology Department, Hassan II University Hospital, Fez, Morocco; 2 Laboratory of Epidemiology and Public Health, Faculty of Medicine and Pharmacy, Sidi Mohammed BenAbdellah university Fez, Morocco; 3 Department of Clinical Neuroscience, Karolinska Institutet, Stockholm, Sweden

**Keywords:** Intravenous thrombolysis, stroke, rt-PA

## Abstract

The majority of strokes are due to blockage of an artery in the brain by a blood clot. Prompt treatment with thrombolytic drugs can restore blood flow before major brain damage has occurred. We report the case series of all patients who were treated with rt-PA at Stroke Unit of HASSAN II University hospital between 2010 and 2013. There were 52 patients treated with intravenous rtPA during the study period. The mean age was 63 years with the no gender predominance (sex ratio 1.02). Hypertension was the most common vascular risk factor (31%) and 17% of patients suffered from atrial fibrillation. 17 of 52 patients (32.7%) were treated within a 3 hours window of stroke onset and 35 of 52 (67.3%) patients were treated within 3-4.5 h. Twenty five patients (48%) had significant early improvements within 24 hours and twenty one (40.3%) patients had good outcomes at 3 months and fifteen patients (29%) died within the same period.

## Introduction

Stroke is one of the leading causes of disability and death in both developing and developed countries. Each year, about 22 million people have a stroke world-wide. While the incidence of stroke in developed countries declined 42% between 1970 and 2008, it doubled in developing countries and exceeded the developed countries between 2000 and 2008 [1, 2]. The prevalence rate of stroke survivors in Morocco is 292/100.000 [3]. Intravenous thrombolytic therapy has been widely recommended as a standard treatment for acute ischemic stroke in most clinical practice guidelines. With the approval of tissue plasminogen activator (rt-PA) for the acute ischemic stroke management, the evolution of this emergency has changed dramatically. However, the number of stroke units and experience on stroke thrombolysis in North Africa is still limited. Lack of infrastructure is main barriers of thrombolysis therapy in developing countries [4, 5]. On 2007, the first stroke unit in Morocco was created in university hospital of Fez, and the first thrombolysis was done on 2010. We report the case series of thrombolytic therapy in Morocco.

## Methods

Patient with acute ischemic stroke treated with intravenous rt-PA at Stroke Unit of HASSAN II University hospital between 2010 and 2013 were included. Intravenous rt-PA was prescribed for acute ischemic stroke within 4.5 hours of onset. Inclusion and exclusion criteria were even that those used in the National Institute of Neurological Disorders and Stroke (NINDS) study [6]. However, in our study older age (more than 80 years old) was not an exclusion criterion. Patients with high blood pressure (systolic blood pressure above 185 mmhg or diastolic blood pressure above 110 mmhg) were not excluded if their blood pressure could be controlled by intravenous nicardipine before rtPA administration.

All patients had pretreatment non contrast CT scans of the brain except for Wake up stroke, where the patients had cerebral MRI. CT scans were evaluated by a neurologist. Informed consent was obtained from all patients, after discussion of the potential benefits and risks, including the risk of symptomatic intracerebral hemorrhage (ICH). Contraindications for thrombolysis included evidence of intracranial hemorrhage, or hypodensity greater than 1/3 of the middle cerebral artery territory in the first year of study. After this period, we used Alberta Stroke Program early CT score (ASPECTS) [7]. Score < 7 was considered as exclusion criteria. The dose of administered rt-PA was 0.9 mg /kg body weight with a maximum of 90 mg. According to protocol, 1/10 of total dose of rt-PA was given intravenously in a bolus and the remaining 9/10 during 1 hour. All Patient treated with intravenous rtPA were admitted to Stroke Unit for clinical monitoring. The initial severity of the stroke and evolution after thrombolysis was evaluated using the National Instituted of Health Stroke Scale (NIHSS). A second cerebral CT scan was performed 24 hours after receiving thrombolysis. Hemorrhagic brain lesion and death were also recorded. Clinical assessment was repeated with NIHSS after 24 hours. Early clinical improvement was defined according to the NINDS criteria as 4-point improvement in the NIHSS score from baseline values or complete resolution of neurological deficit. Significant clinical deterioration was defined as 4-point NIHSS deterioration at 24 hours. The modified Rankin scale (mRS) was evaluated after 90 days. The outcome was considered as good outcome if mRS at 3 months was between 0 and 2. Symptomatic intracranial hemorrhage was defined according to ECASS III as any hemorrhage with neurologic deterioration combined with an NIHSS score 4 points greater than either the baseline value or the lowest value in the first 7 days, or death [8].

All patients were registered in the International Stroke Thrombolysis Register (ISTR) to compare our results with other centers worldwide [9]. It is an internet-based, international monitoring registry for auditing the safety and efficacy of routine therapeutic use of thrombolysis in acute ischemic stroke. To evaluate difference in prognosis between the first period of starting thrombolysis in our center without using ASPECTS and second period when we use it, we divided the group on two subgroups. First subgroup from April 2010 to December2011, the second from January 2012 to December 2013.

## Results

There were 52 patients treated with intravenous rtPA during the study period. The demographic characteristics, vascular risk factors, stroke subtype, and baseline clinical parameters are given in Table 1. The mean age was 63 years with the no gender predominance (sex ratio 1.02). Hypertension was the most common vascular risk factor (31%) and 17% of patients suffered from atrial fibrillation. 17 of 52 patients (32.7%) were treated within a 3 hours window of stroke onset and 35 of 52 (67.3%) patients were treated within 3-4.5 h. Mean door-to-needle time was 75 min. Mean onset-to treatment time (OTT) was 212 minutes (Table 2). Systolic and diastolic blood pressures at admission were 139 mmHg and 78 mmHg respectively. Initial median NIHSS score was 14. The initial NIHSS was more severe in the first subgroup (NIHSS> 15 in 58 % of patients) than in the second subgroup (NIHSS> 15 in only 28 % of patients) (Table 3). Median NIHSS changes 2 hours post thrombolysis was -3. Median NIHSS changes 24 hours post thrombolysis was -2. There were three asymptomatic ICH (5,7%) and four symptomatic ICHs (7,7%). Two of the four symptomatic ICHs were fatal. Four patients had minimal gingival hemorrhages and only one case of hematuria without haemodynamic compromise. Twenty five patients (48%) had significant early improvements within 24 hours and twenty one (40,3%) patients had good outcomes at 3 months and fifteen patients (29%) died within the same period (Table 4). The causes of death were cerebral infarct (4/15: 26,6%), acute myocardial infarction (1/15: 6,6%), intracerebral hemorrhage (2/15:13,3%), unknown causes (4/15: 26,6%) and other cause (4/15: 26,6%).

**Table 1:** Demographic and baseline clinical characteristics

Baseline characteristics	Morocco center
**Age (Mean)**	
**Female (%)**	
**Male (%)**	
**Risk factors**	
Hypertension	31%
Diabetes	12%
Hyperlipidaemia	8%
Curent smoker	4%
Previous smoker	
Previous clin. diag. ischaemic stroke (earlier than 3 m)	4%
Previous clin. diag. ischaemic stroke (within 3 m)	2%
Atrial fibrillation	17%
Congestive heart failure	10%
**Baseline NIHSS**	
Median NIHSS	14.0
NIH LEVEL 0-7	2%
NIH LEVEL8-14	**52%**
NIH LEVEL 15PLUS	**46%**
**Ischemic Stroke Subgroups**	
I63.0: Cerebral infarct, large vessel disease with significant carotid stenosis (>50% NASCET)	9%
I63.3: Cerebral infarct, other large vessel disease	23%
I63.4: Cerebral infarct, cardiac emboli	50%
I63.5: Cerebral infarct, small vessel/lacunar	0%
I63.6: Cerebral infarct, sinus venous thrombosis	0%
I63.8: Cerebral infarct, other/ unusual cause	0%
I63.9: Cerebral infarct, multiple/ unknown cause	18%

**Table 2:** Time delay and comparison with SITS ISTR results

Time delay (minutes)	Morocco Center	Within 3 hours SITS-ISTR	Within 3 to 4.5hours SITS-ISTR
Onset to treating hospital/door time(median)	120.0	65.0	130.0
Door to treatment/needle time (median)	75.0	65.0	80
Onset to treatment/needle time (median)	209.0	138.0	210

**Table 3:** Initial NIHSS in the 2 subgroups

	2010-2011	2012-2013
Median NIHSS	15	14
NIH LEVEL0-7	3%	0%
NIH LEVEL8-14	**39%**	**72%**
NIH LEVEL15 Plus	**58%**	**28%**

**Table 4:** Clinical outcome details

Clinical outcome details	Morocco Center
**NIHSS changes within 24h**	
Change in NIHSS 0-2h (median)	-3.0
Change in NIHSS 0-24h (median)	-2.0
**Significant early improvements**	**48%**
Significant deterioration	29%
**Rankin 3 months**	
0 No symptoms at all	16%
1 No significant disabling symptoms	18%
2 Slight disability	6%
3 Moderate disability	6%
4 Moderate severe disability	24%
5 Severe disability	2%
6 Dead	29%
**Global Outcome (24h)**	
Much better	17%
Better	33%
Unchanged	23%
Worse	12%
Much worse	6%
Dead	10%

## Discussion

We analyzed the data of 52 thrombolysed patients gathered over a 3years period. In developing countries like morocco, only a small proportion of patients with ischemic stroke currently receive the thrombolytic therapy with IV rt-PA. The reason for this include the scarcity of stroke units, in fact our stroke unit in Hassan II Hospital University is the only one in Morocco. Difficulties in patient recognition of stroke symptoms, delays in seeking appropriate emergency care, delays in obtaining urgent brain imaging scan and also cost constraint, indeed the rt-PA is not covered by medical insurance in morocco are the others reasons [4]. The important delay to admission at hospital (120 minutes) compared with SITS delay (65 minutes) was explained by the fact that all patients are transported on their own: car, taxi, private ambulance. This is due to lack of organization providing such transportation [10]. The lack of prehospital organization and lack of people awareness about stroke are the most frequent explanation in developing countries. Compared with SITS-ISTR, mean time to door is about 55 min longer in our serie than in the cohort of patients treated within 3 hours of onset and 10 min under in the cohort of patients treated within 3 to 4.5 hours. The median time delay from hospital admission to initiation of treatment in our center was 10 minutes longer in the 3 hours cohort and 5 minutes under in the 3- to 4.5-hour cohort [10].

**Figure 1:**
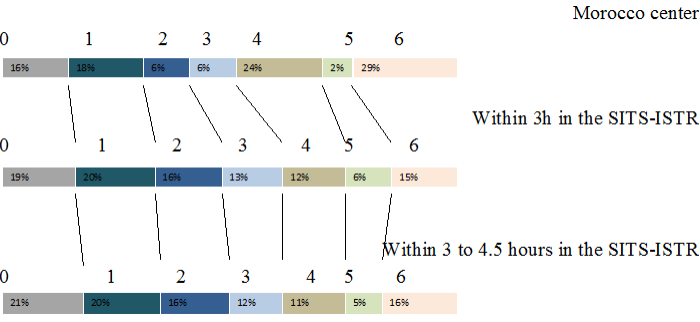
Comparison of mRS at 3 months between Moroccan patients, patients treated within 3–4.5 h and within 3 h in the SITS-ISTR

Before we started performing rt-PA, we performed a series of teaching courses, to educate physicians in local emergencies on the importance of early recognition and treatment of stroke patients. We educated radiologists and laboratory personnel in importance of early and fast work-up of stroke patients, and that patient represents a true neurological emergency. These teaching courses resulted in acceptable door to needle time in our hospital. Our future plans include providing more effective public education and working closely with paramedical personnel to shorten the time between the symptom onset and arrival to the emergency department. A Portuguese study found that the pre-hospital care account for 82% of the total delay of care. It is especially at this level that we must also act to guide the patient directly to the hospital [11]. The group of independent patients at 3 months is 39% in our series (20 cases) compared to 55% in all patients treated within 3 hours and within 3 to 4.5 hours in the SITS-ISTR (Figure 1).

At the 3-month follow-up, only 21 patients (40%) were functionally independent (mRS ≤2). The death at 3 months in our study is significantly higher than in SITS-ISTR centers (29% compared to 16% for participants in 3 to 4.5 hours in the SITS-ISTR centers). The meta-analysis of Graham and Al. with 15 open studies (2639 patients) found a death rate of 13% [12]. The analysis of these results must take into account the initial NIHSS score is an average of 14 in our series compared with 09 in 3 to 4.5 hours cohort in the SITS-ISTR [10]. In addition, the percentage of patients with an initial thrombolysis NIHSS score less than 7 represents only 2% in our series. Another factor must also be taken into account in our context: the onset to treatment/needle time, thrombolysis patients in our series before 3 hours are only 26%, this explain the results very close to our center with 3 to 4.5hours cohort in the SITS-ISTR. Furthermore, the individual review of patient records included in the NINDS, ECASS I study and II, ATLANTIS (included patients until 6 assigns tests) showed that the positive effect in terms of deaths and dependence treatment was inversely correlated with the time of treatment after the onset of symptoms, noting that the risk-benefit ratio is favorable up to 4h 30 min [13]. In a 2009 Cochrane Systemic Review, symptomatic ICH was found in 7.7%, and death occurred in 16.5%, of the patients treated with thrombolysis [14]. Compared to the NINDS study and 2009 Cochrane data, a slightly lower symptomatic ICH and mortality rate were observed in our study. However, patients in the NINDS study were treated within three hours after the onset of stroke, which differed from our study. Patients with either symptomatic or asymptomatic ICH had less favorable outcomes and higher mortality rates compared to patients without ICH.

To improve our results, we must develop our multidisciplinary team of emergency physicians and nurses, radiologists, laboratory and paramedical personnel, and stroke neurologists for implementing the fast-track approach to providing thrombolysis. More rapid identification and evaluation in the emergency department, diagnosis, and initiation of treatment also could be responsible for the lower rate of SICH and better outcomes. Entry of the data for all our rt-PA-treated patients into the ongoing SITS multinational stroke registry (https://sitsinternational.org) is important initiative to ensure that our process and outcome measures in stroke thrombolysis are comparable to those in other international centers.

## Conclusion

In, conclusion we have been able to show that IV thrombolysis feasible and safe in our hospital. This calls for a vigorous campaign among the public, health workers to develop other stroke unit.

### What is known about this topic

- Stroke is one of the leading causes of disability and death in both developing and developed countries;
- The number of stroke patients receiving r-tPA in the developing world is extremely low;
- The chief benefit of thrombolysis is improved final functional outcome through reperfusion salvage of threatened tissue.

### What this study adds

- The interest of this study is to bring our experience of 52 patients to encourage the realization of thrombolysis in Africa;
- Increase the number of stroke units and the patients receiving r tPA in the developing countries;
- Improving the management of ischemic stroke.
